# Supplementary figures and images for: Does joint impedance improve dynamic leg simulations with explicit and implicit solvers?
Source: PLoS One. 2023 Jul 3;18(7):e0282130. doi: 10.1371/journal.pone.0282130 (PMC10317227; doi:10.1371/journal.pone.0282130)

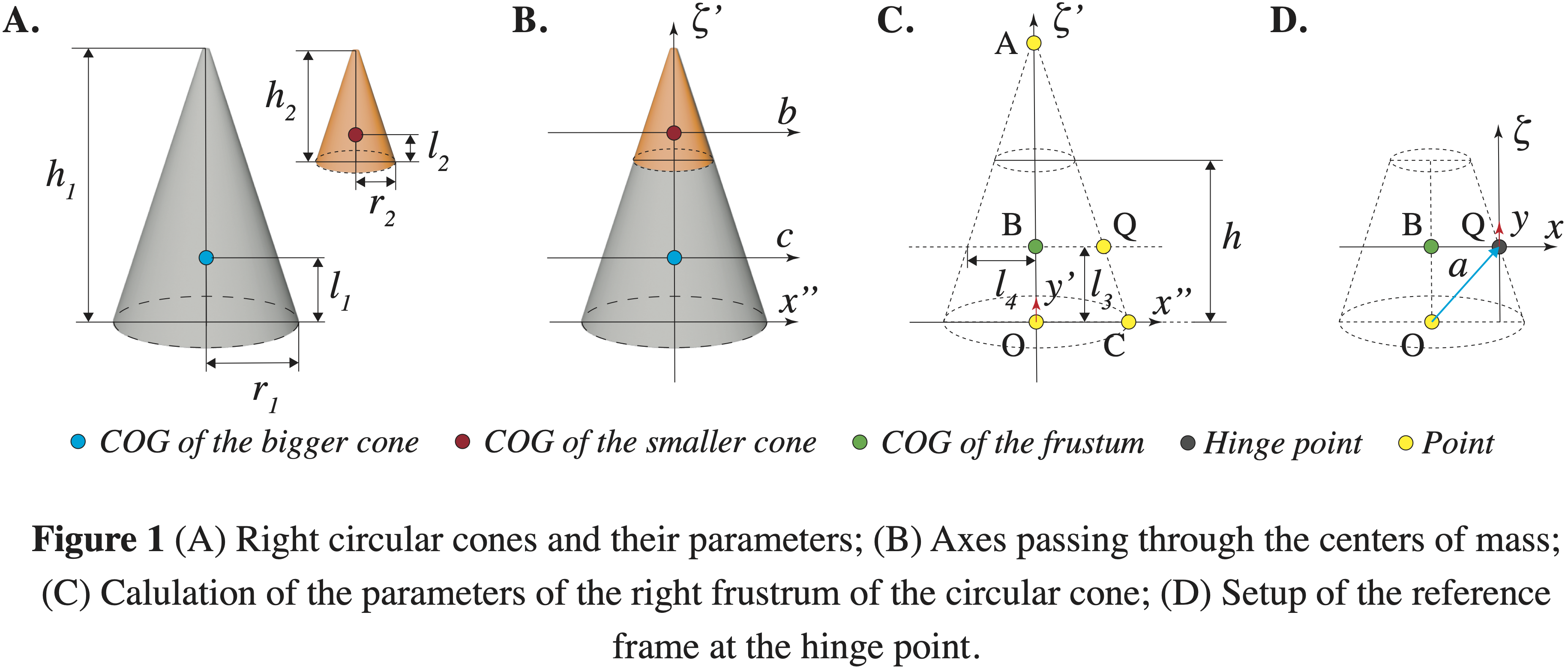

Supplement: S1 Fig — (A) Dimensions and centers of mass of the right circular cones; (B) secondary axes used to derive inertia of the frustum; (C) frustum dimensions used to set up a reference frame; (D) frustum modeling human foot with the reference frame originating at the ankle joint. (PNG) [file pone.0282130.s001.png]

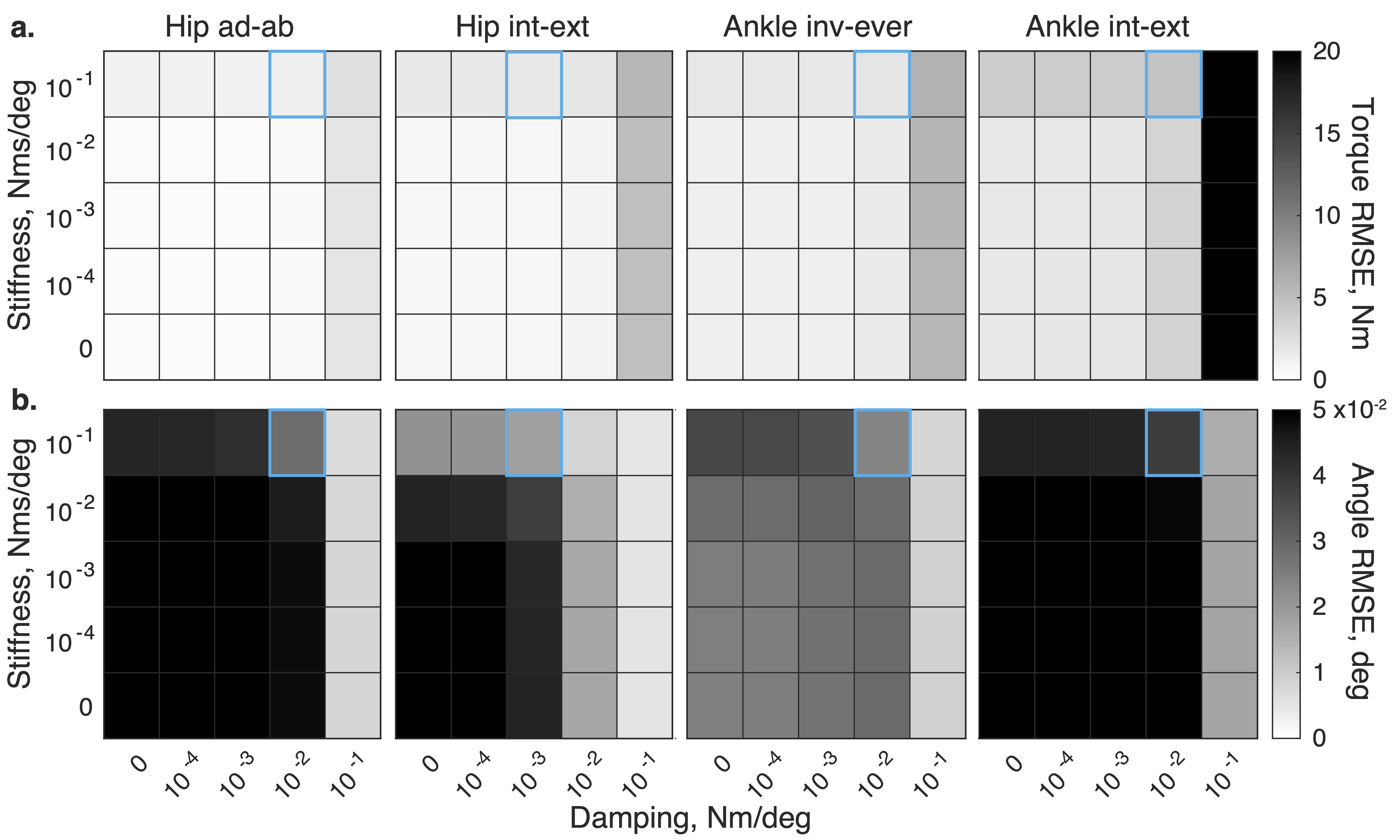

Supplement: S2 Fig — Kinetic (a) and kinematic (b) errors are affected by stabilizing impedance differently. The errors are shown for the hip adduction-abduction, hip internal external rotation, ankle inversion-eversion, and ankle internal external rotations DOFs of the swinging leg during one representative swing phase simulated with the implicit Euler method at 200 Hz. The kinetic performance decreases at high values, and the kinematic performance decreases at low values. The errors obtained with optimal (k,b), specific to DOF, sampling frequency, and solver, are squared in light blue. Abbreviations: ad-ab—adduction-abduction; int-ext—internal-external rotation; inv-ever—inversion-eversion. (PNG) [file pone.0282130.s002.png]

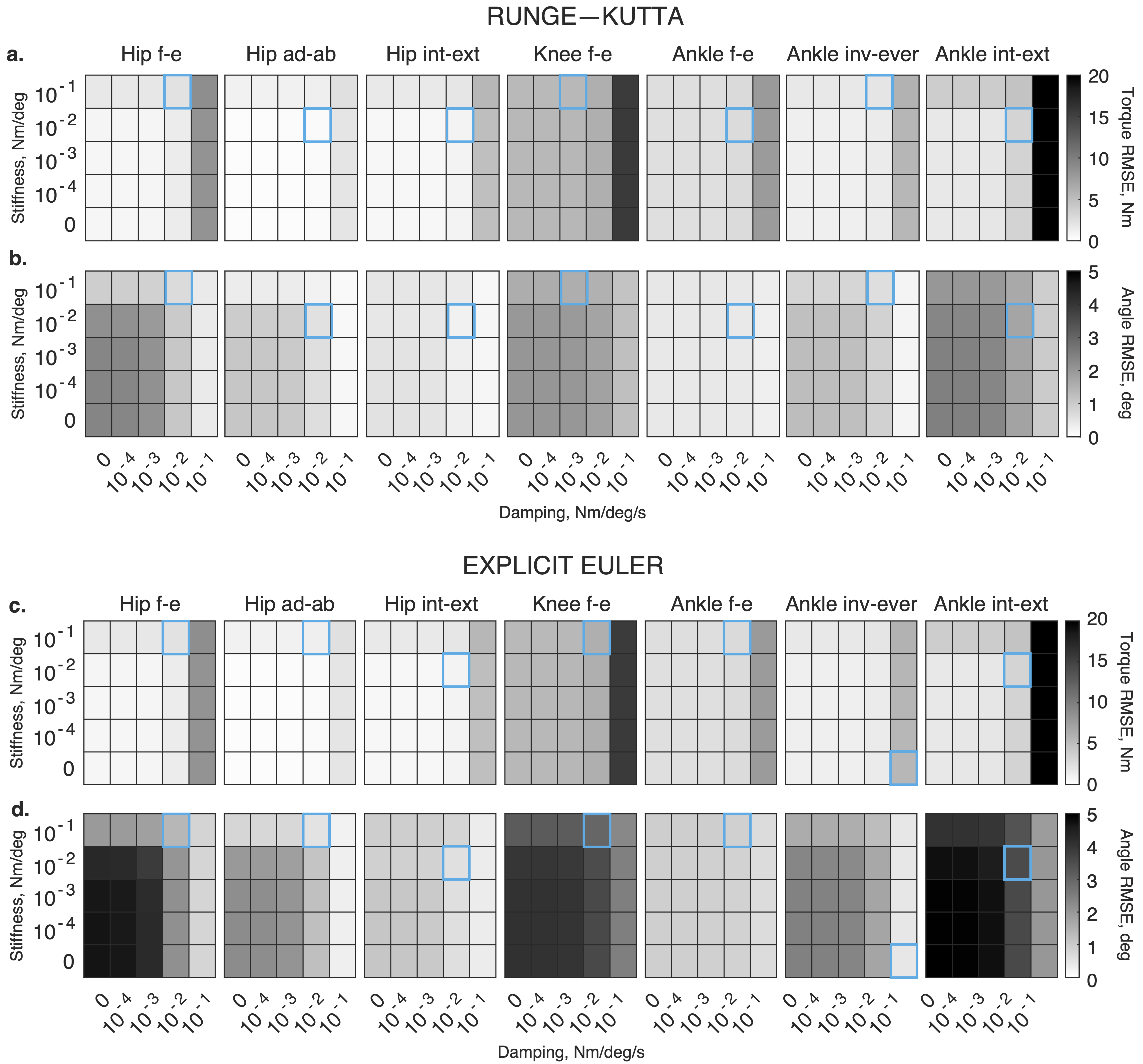

Supplement: S3 Fig — Stabilizing impedance has dissimilar effects on (a,c) kinetic and (b,d) kinematic errors across DOFs and solver types. The errors are shown for the hip flexion-extension, hip adduction-abduction, hip internal-external rotation, knee flexion-extension, ankle flexion-extension, ankle inversion-eversion, and ankle internal-external rotations DOFs of the swinging leg during one representative swing phase simulated with the built-in recursive (a,c), 4th order Runge-Kutta (b), and explicit Euler (d) methods at 200 Hz. The kinetic performance decreases at high values, and the kinematic performance decreases at low values. The errors obtained with optimal (k,b), specific to DOF, sampling frequency, and solver, are squared in light blue. Notice a hundredfold difference in scale between values in Fig 2B and S2B Fig. Abbreviations: f-e—flexion-extension; ad-ab—adduction-abduction; int-ext—internal-external rotation; inv-ever—inversion-eversion. (PNG) [file pone.0282130.s003.png]

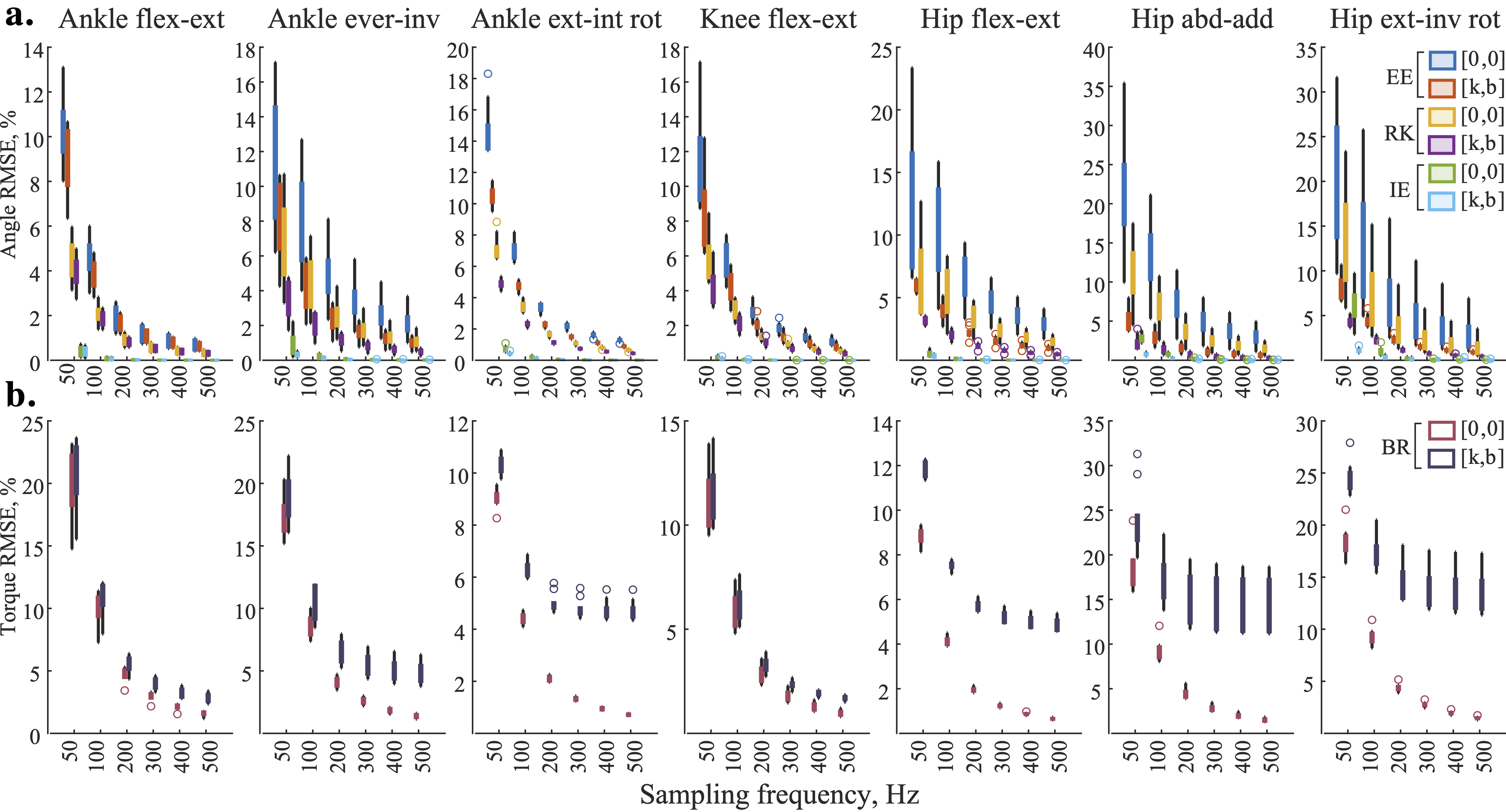

Supplement: S4 Fig — Forward (A) and inverse (B) simulations accuracy are shown as a function of sampling rate, viscoelastic contribution, and numerical solver. The simulations in (A) were solved using numerical integrators: EE—explicit Euler method, RK—4th order Runge-Kutta method, and IE—implicit Euler method. Corresponding inverse simulations (B) were solved with a built-in recursive solver—BR. Labels [k,b] marked the error distributions obtained with optimal impedance. (PNG) [file pone.0282130.s004.png]
