# Supplementary material for: Does joint impedance improve dynamic leg simulations with explicit and implicit solvers?
Source: PLoS One. 2023 Jul 3;18(7):e0282130. doi: 10.1371/journal.pone.0282130 (PMC10317227; doi:10.1371/journal.pone.0282130)
Supplement: S1 File — (DOCX) [file pone.0282130.s005.docx]

# **Supplementary Information**

## **Deriving body inertia using Hannavan’s formulations**

We modeled the thigh, shank, and foot segments using the frusta of the right circular cones. As an example, we demonstrate the computations performed for the foot segment. We employed similar equations to derive the thigh and shank segments’ inertia.


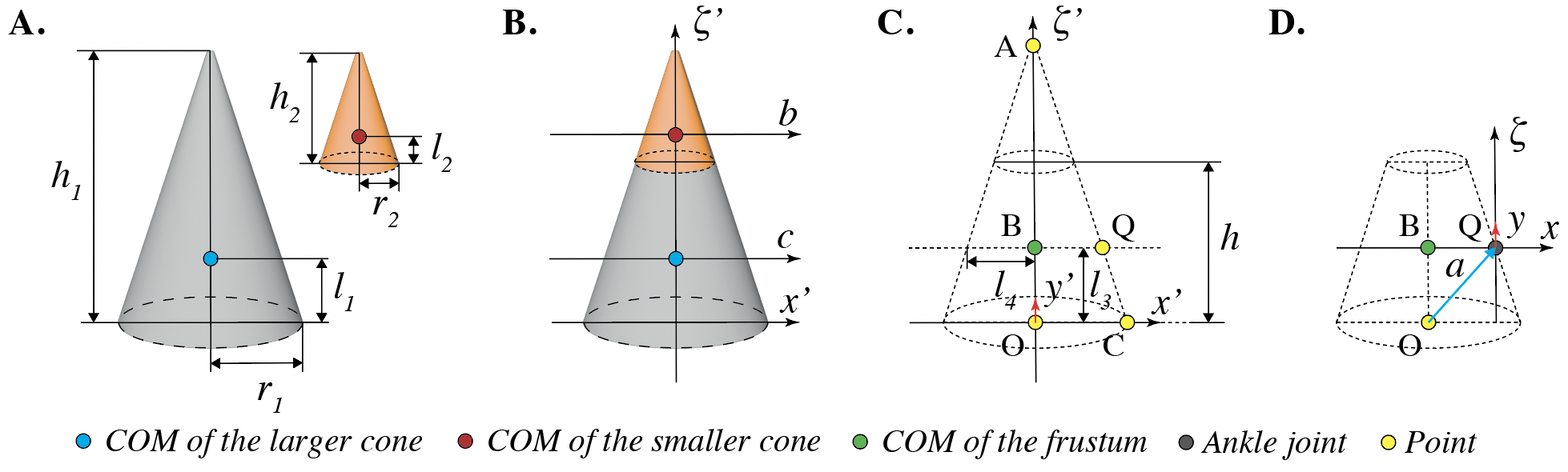


**S1 Fig.** Mathematical model of the human foot inertia. (A) Dimensions and centers of mass of the right circular cones; (B) secondary axes used to derive inertia of the frustum; (C) frustum dimensions used to set up a reference frame; (D) frustum modeling human foot with the reference frame originating at the ankle joint.

The inertial properties of the right circular cone describing the foot segment (Fig. S1D) are calculated as a difference between the inertia of the larger cone of height $h_{1}$ and inertia of the smaller cone of height $h_{2}$ (Fig. S1A). From Fig. S1B, the inertia moment of the larger cone about the axis $c$, passing through its center of mass, is equal to:

| $I_{c}=\frac{3}{20}M_{1}(r_{1}^{2}+ \frac{h_{1}^{2}}{4})$, | (1) |
| --- | --- |

where $M_{1}$ is the mass of the larger cone and $r_{1}$ is the radius of its proximal base.

This equation can be simply modified to describe the larger cone’s inertia around the $x’$ axis using the parallel axis theorem (PAT). In its general form, PAT can be written as:

| $J_{ij}= I_{ij}+M (a^{2}\delta_{ij}- a_{i}a_{j})$, | (2) |
| --- | --- |

where $I_{ij}$ is an element of the $i^{th}$ row and $j^{th}$ column of the inertia matrix, defined with respect to the center of mass; $J_{ij}$ is an element of the inertia matrix defined with respect to the new reference frame, $M$ is the mass of the solid body; $a$ is a vector pointing from the body’s center of mass to the origin of the new reference frame; $\delta$ is called Kronecker delta and is equal to 1 if $i = j$ and to 0 if $i \neq j$.

Thus, inserting eq. 1 in eq. 2 produces larger cone’s inertia calculated with respect to the $x’$:

| $I_{x_{b}^{'}}=I_{c}+ M_{1}l_{1}^{2}$, | (3) |
| --- | --- |

where $l_{1}=h_{1}/4$ is the distance from the base of the larger cone to its center of mass.

The same calculations can be performed for the smaller cone of the height $h_{2}$. The inertia moment around the axis $b$, passing through the center of mass, is equal to:

| $I_{b}=\frac{3}{20}M_{2}(r_{2}^{2}+ \frac{h_{2}^{2}}{4})$, | (4) |
| --- | --- |

where $M_{2}$ is the mass of the smaller cone and $r_{2}$ is the radius of its proximal base.

Consequently, inserting eq 4. in eq. 2 produces the moment of inertia of the smaller cone around the $x’$ axis:

| $I_{x_{s}^{'}}=I_{b}+ M_{2}{(l_{2}+h)}^{2}$, | (5) |
| --- | --- |

where $l_{2}=h_{2}/4$ is the distance from the base of the larger cone to its center of mass, $h= {h_{1}- h}_{2}$ is a height of the frustum shown in Fig. S1C.

Notably, masses $M_{1}$ and $M_{2}$ used when computing inertia matrix cannot be found in anthropometric reference tables; thus, these were computed assuming uniform distribution of the cones and frustum’s density:

*(i) larger cone’s volume*

| $V_{1}= \frac{1}{3}\pi r_{1}^{2}h_{1}$ | (6.1) |
| --- | --- |

*(ii) smaller cone’s volume*

| $V_{2}= \frac{1}{3}\pi r_{2}^{2}h_{2}$ | (6.2) |
| --- | --- |

*(iii) frustum’s volume*

| $V=V_{1}-V_{2}$ | (6.3) |
| --- | --- |

*(iv) cones’ masses*

| $\rho=\frac{M}{V} \Rightarrow\left\vert\begin{matrix} M_{1}= \rho V_{1} \\ M_{2}= \rho V_{2} \end{matrix} \right.$ | (6.4) |
| --- | --- |

In addition, the heights of the cones and the frustum relate as follows (Fig. S1):

| $\frac{h_{1}}{r_{1}}= \frac{h_{2}}{r_{2}}= \frac{h}{{r_{1}-r}_{2}}\Rightarrow h_{1}= \frac{r_{1}h}{{r_{1}-r}_{2}}, h_{2}= \frac{r_{2}h}{{r_{1}-r}_{2}}$ | (7) |
| --- | --- |

As a result, the moment of inertia describing the frustum is computed by the difference in the inertia moments of the two cones about the $x’$ axis:

| $I_{x^{'}}=I_{x_{b}^{'}}- I_{x_{s}^{'}}$ | (8) |
| --- | --- |

Due to the circular symmetricity of the frustum, its rotational inertia around $y'$ axis (Fig. S1C) is equal to the inertia around $x’$ axis:

| $I_{y^{'}}= I_{x^{'}}$ | (9) |
| --- | --- |

Rotation of the frustum around $\zeta^{'}$ axis with a center of rotation set in the point $O$ (Figure S1C) can be similarly found by subtracting inertias of the two cones:

*(i) larger cone*

| $I_{\zeta_{b}^{'}}=\frac{3}{10}M_{1}r_{1}^{2}$, | (10) |
| --- | --- |

*(ii) smaller cone*

| $I_{\zeta_{s}^{'}}=\frac{3}{10}M_{2}r_{2}^{2}$, | (11) |
| --- | --- |

*(iii) frustum*

| $I_{\zeta^{'}}=I_{\zeta_{b}^{'}}- I_{\zeta_{s}^{'}}$ | (12) |
| --- | --- |

Therefore, frustum’s matrix of inertia moments with respect to $x’$-$y'$*-*$\zeta^{'}$reference frame is defined as follows:

| $I= \left[ \begin{matrix} I_{x^{'}} & 0 & 0 \\ 0 & I_{y^{'}} & 0 \\ 0 & 0 & I_{\zeta^{'}} \end{matrix} \right]$ | (13) |
| --- | --- |

To describe foot inertia with respect to the $x$-$y$-$\zeta$ frame (foot coordinate system) originating in the point $Q$ (ankle joint, Fig. S1D), eq. 10 needs to be further modified using PAT. Thus, we define the coordinates of the vector pointing from the origin of the frame $O$ to the point $Q$: $a (l_{4}, 0, l_{3})$. Segment $OB= l_{3}$ defines the coordinate of the frustum’s centroid along the $\zeta^{'}$ axis; thus, $l_{3}$ can be found from the equation for the centroid:

| $l_{3}=\frac{h}{4}\cdot\frac{r_{1}^{2}+2r_{1}r_{2}+3r_{2}^{2}}{r_{1}^{2}+r_{1}r_{2}+r_{2}^{2}}$ | (14.1) |
| --- | --- |

The length of the segment $BQ=l_{4}$ can be found from the similar triangles $\Delta AOC$ and $\Delta ABQ$:

| $\frac{AO}{AB}= \frac{OC}{BQ} \Rightarrow\frac{h_{1}}{h_{1}- l_{3}}= \frac{r_{1}}{l_{4}} \Rightarrow l_{4}= \frac{r_{1}(h_{1}- l_{3})}{h_{1}}$ | (14.2) |
| --- | --- |

Therefore, applying eq. 2 to eq. 13 produces a matrix describing the inertial properties of the foot segment with respect to the foot coordinate system with the origin in the ankle joint:

| $J= \left[ \begin{matrix} I_{x^{'}}+M\left( \left[ l_{4}^{2}+ l_{3}^{2} \right]- l_{4}^{2} \right) & 0 & -Ml_{3}l_{4} \\ 0 & I_{y^{'}}+M\left( l_{4}^{2}+ l_{3}^{2} \right) & 0 \\ -Ml_{3}l_{4} & 0 & I_{\zeta^{'}}+M\left( \left[ l_{4}^{2}+ l_{3}^{2} \right]- l_{3}^{2} \right) \end{matrix} \right]$ | (15) |
| --- | --- |

## **Viscoelastic impedance**

S1 Table. The sampling rate and numerical integrator choice influence optimal stiffness across DOF. Stiffness values are shown in Nm/deg. Abbreviations: f-e—flexion-extension; ab-ad—abduction-adduction; int-ext—internal-external rotation; ever-inv—eversion-inversion; EE—explicit Euler method; RK—4^th^ order Runge-Kutta method; IE—implicit Euler method.

|  |  | Sampling rate, Hz | | | | | | | | | | | | | | | | | |
| --- | --- | --- | --- | --- | --- | --- | --- | --- | --- | --- | --- | --- | --- | --- | --- | --- | --- | --- | --- |
|  |  | 50 | | | 100 | | | 200 | | | 300 | | | 400 | | | 500 | | |
| Joint | DOF | EE | RK | IE | EE | RK | IE | EE | RK | IE | EE | RK | IE | EE | RK | IE | EE | RK | IE |
| Ankle | flex-ext | 0 | 10^-1^ | 10^-1^ | 10^-3^ | 10^-1^ | 10^-1^ | 10^-1^ | 10^-2^ | 10^-1^ | 10^-2^ | 10^-2^ | 10^-1^ | 10^-2^ | 10^-2^ | 10^-1^ | 10^-2^ | 10^-2^ | 10^-1^ |
|  | ever-inv | 10^-1^ | 10^-1^ | 10^-1^ | 10^-2^ | 0 | 10^-1^ | 0 | 10^-1^ | 10^-1^ | 10^-1^ | 10^-1^ | 10^-1^ | 10^-2^ | 10^-2^ | 0 | 10^-2^ | 10^-2^ | 10^-4^ |
|  | int-ext rot | 10^-1^ | 10^-1^ | 10^-1^ | 10^-1^ | 10^-1^ | 10^-1^ | 10^-2^ | 10^-2^ | 10^-1^ | 10^-2^ | 10^-2^ | 10^-1^ | 10^-2^ | 10^-2^ | 10^-1^ | 10^-2^ | 10^-2^ | 10^-1^ |
| Knee | flex-ext | 10^-1^ | 10^-1^ | 10^-1^ | 10^-1^ | 10^-1^ | 0 | 10^-1^ | 10^-1^ | 10^-1^ | 10^-1^ | 10^-1^ | 10^-1^ | 10^-1^ | 10^-1^ | 10^-1^ | 10^-1^ | 10^-1^ | 10^-1^ |
| Hip | flex-ext | 10^-1^ | 10^-1^ | 10^-2^ | 0 | 10^-1^ | 10^-1^ | 10^-1^ | 10^-1^ | 10^-4^ | 10^-1^ | 10^-1^ | 0 | 10^-1^ | 10^-1^ | 0 | 10^-1^ | 10^-1^ | 10^-3^ |
|  | abd-add | 10^-2^ | 10^-1^ | 10^-1^ | 10^-1^ | 10^-1^ | 10^-1^ | 10^-1^ | 10^-2^ | 10^-1^ | 10^-1^ | 10^-2^ | 10^-1^ | 10^-2^ | 10^-2^ | 10^-1^ | 10^-2^ | 10^-2^ | 10^-1^ |
|  | int-ext rot | 0 | 0 | 10^-1^ | 0 | 10^-2^ | 10^-1^ | 10^-2^ | 10^-2^ | 10^-1^ | 10^-2^ | 10^-2^ | 10^-1^ | 10^-2^ | 10^-2^ | 10^-1^ | 10^-2^ | 10^-2^ | 10^-1^ |

S2 Table. The sampling rate and numerical integrator choice influence optimal damping across DOF. Damping values are shown in Nm/deg/s. Abbreviations are the same as in Table S1.

|  |  | Sampling rate, Hz | | | | | | | | | | | | | | | | | |
| --- | --- | --- | --- | --- | --- | --- | --- | --- | --- | --- | --- | --- | --- | --- | --- | --- | --- | --- | --- |
|  |  | 50 | | | 100 | | | 200 | | | 300 | | | 400 | | | 500 | | |
| Joint | DOF | EE | RK | IE | EE | RK | IE | EE | RK | IE | EE | RK | IE | EE | RK | IE | EE | RK | IE |
| Ankle | flex-ext | 10^-1^ | 10^-1^ | 0 | 10^-1^ | 10^-2^ | 0 | 10^-2^ | 10^-2^ | 10^-3^ | 10^-2^ | 10^-2^ | 0 | 10^-2^ | 10^-2^ | 10^-4^ | 10^-2^ | 10^-2^ | 10^-3^ |
|  | ever-inv | 10^-1^ | 10^-1^ | 10^-2^ | 10^-1^ | 10^-1^ | 10^-2^ | 10^-1^ | 10^-2^ | 10^-2^ | 10^-2^ | 10^-2^ | 10^-2^ | 10^-2^ | 10^-2^ | 0 | 10^-2^ | 10^-2^ | 0 |
|  | int-ext rot | 10^-1^ | 10^-1^ | 10^-2^ | 10^-1^ | 10^-2^ | 10^-2^ | 10^-2^ | 10^-2^ | 10^-2^ | 10^-2^ | 10^-2^ | 10^-2^ | 10^-2^ | 10^-2^ | 10^-2^ | 10^-2^ | 10^-2^ | 10^-2^ |
| Knee | flex-ext | 10^-1^ | 10^-1^ | 10^-2^ | 10^-1^ | 10^-2^ | 10^-3^ | 10^-2^ | 10^-3^ | 0 | 10^-3^ | 10^-3^ | 0 | 10^-3^ | 10^-3^ | 10^-4^ | 10^-3^ | 10^-3^ | 0 |
| Hip | flex-ext | 10^-1^ | 10^-1^ | 10^-2^ | 10^-1^ | 10^-2^ | 10^-2^ | 10^-2^ | 10^-2^ | 10^-3^ | 10^-2^ | 10^-2^ | 10^-2^ | 10^-2^ | 10^-2^ | 0 | 10^-2^ | 10^-2^ | 0 |
|  | abd-add | 10^-1^ | 10^-1^ | 10^-2^ | 10^-1^ | 10^-2^ | 10^-2^ | 10^-2^ | 10^-2^ | 10^-2^ | 10^-2^ | 10^-2^ | 10^-3^ | 10^-2^ | 10^-2^ | 10^-4^ | 10^-2^ | 10^-2^ | 10^-4^ |
|  | int-ext rot | 10^-1^ | 10^-1^ | 10^-2^ | 10^-1^ | 10^-2^ | 10^-3^ | 10^-2^ | 10^-2^ | 10^-3^ | 10^-2^ | 10^-2^ | 10^-3^ | 10^-2^ | 10^-2^ | 0 | 10^-2^ | 10^-2^ | 0 |

Forward and inverse simulations


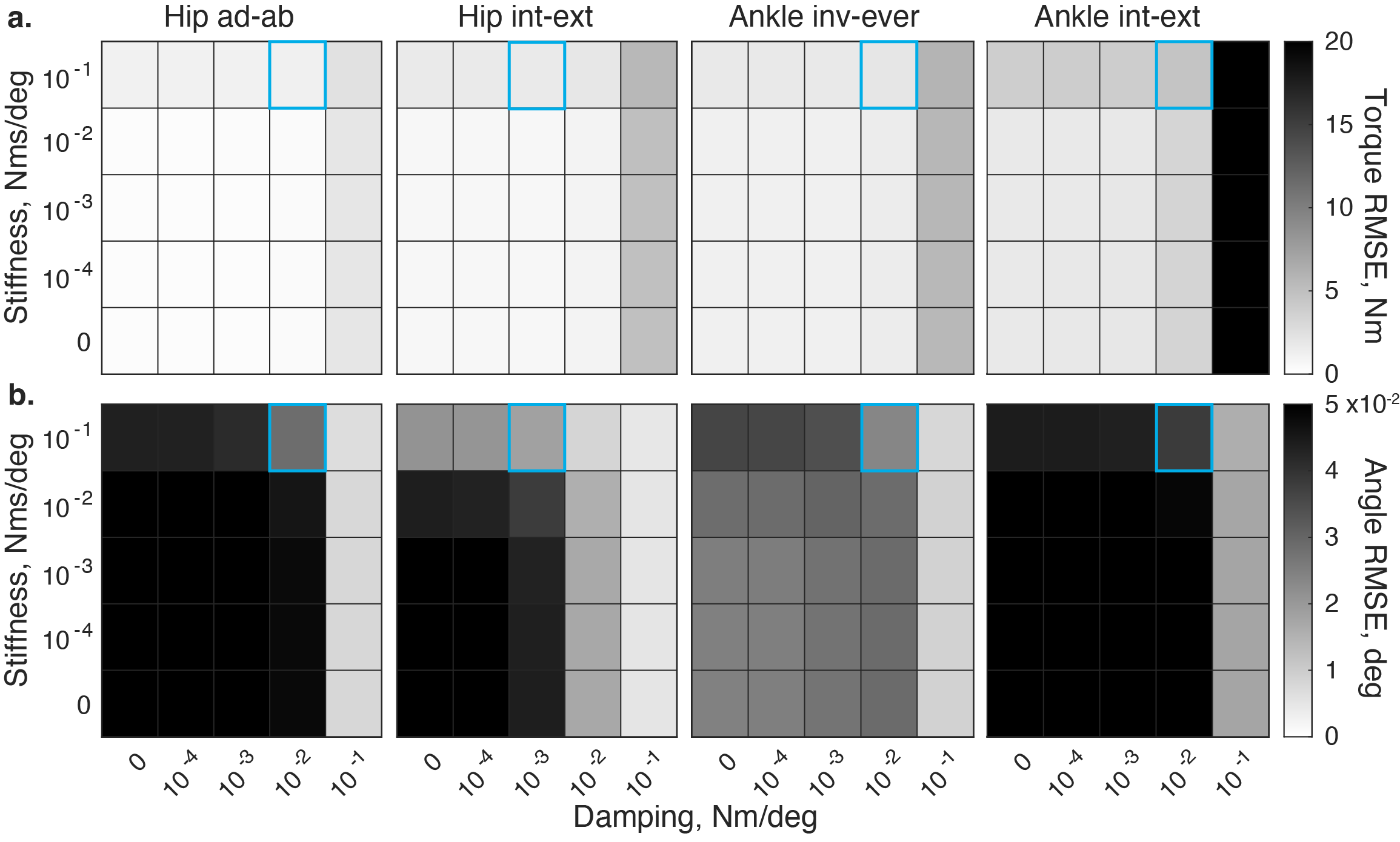


**S2 Fig.**  **Kinetic (a) and kinematic (b) errors are affected by stabilizing impedance differently**. The errors are shown for the hip adduction-abduction, hip internal external rotation, ankle inversion-eversion, and ankle internal external rotations DOFs of the swinging leg during one representative swing phase simulated with the implicit Euler method at 200 Hz. The kinetic performance decreases at high values, and the kinematic performance decreases at low values. The errors obtained with optimal (*k,b*), specific to DOF, sampling frequency, and solver, are squared in light blue. Abbreviations: ad-ab—adduction-abduction; int-ext—internal-external rotation; inv-ever— inversion-eversion.


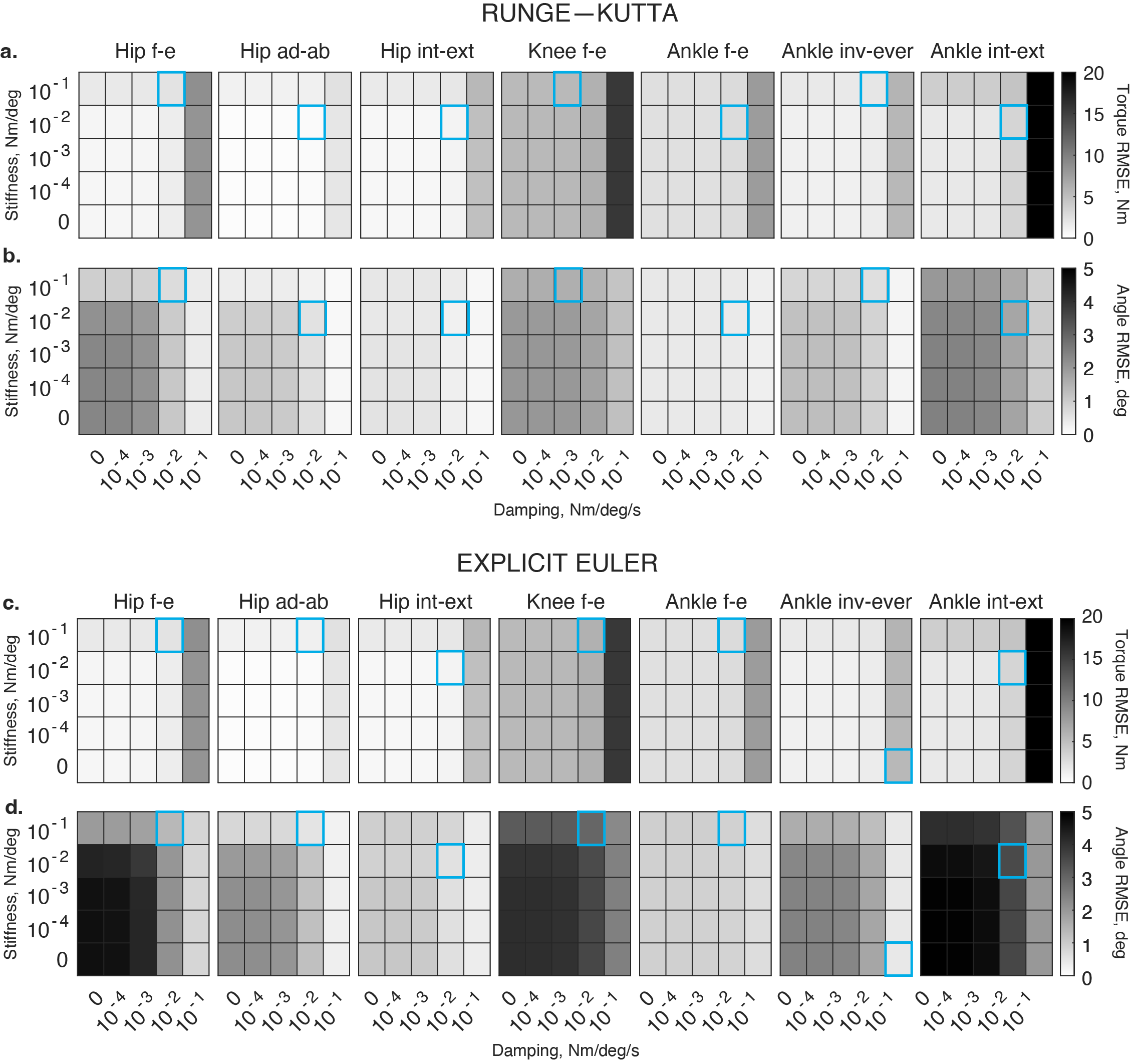


**S3 Fig.**  **Stabilizing impedance has dissimilar effects on (a,c) kinetic and (b,d) kinematic errors across DOFs and solver types.** The errors are shown for the hip flexion-extension, hip adduction-abduction, hip internal-external rotation, knee flexion-extension, ankle flexion-extension, ankle inversion-eversion, and ankle internal-external rotations DOFs of the swinging leg during one representative swing phase simulated with the built-in recursive (a,c), 4^th^ order Runge-Kutta (b), and explicit Euler (d) methods at 200 Hz. The kinetic performance decreases at high values, and the kinematic performance decreases at low values. The errors obtained with optimal (*k,b*), specific to DOF, sampling frequency, and solver, are squared in light blue. Notice a hundredfold difference in scale between values in Figs 2b and S2b. Abbreviations: f-e—flexion-extension; ad-ab—adduction-abduction; int-ext—internal-external rotation; inv-ever— inversion-eversion.


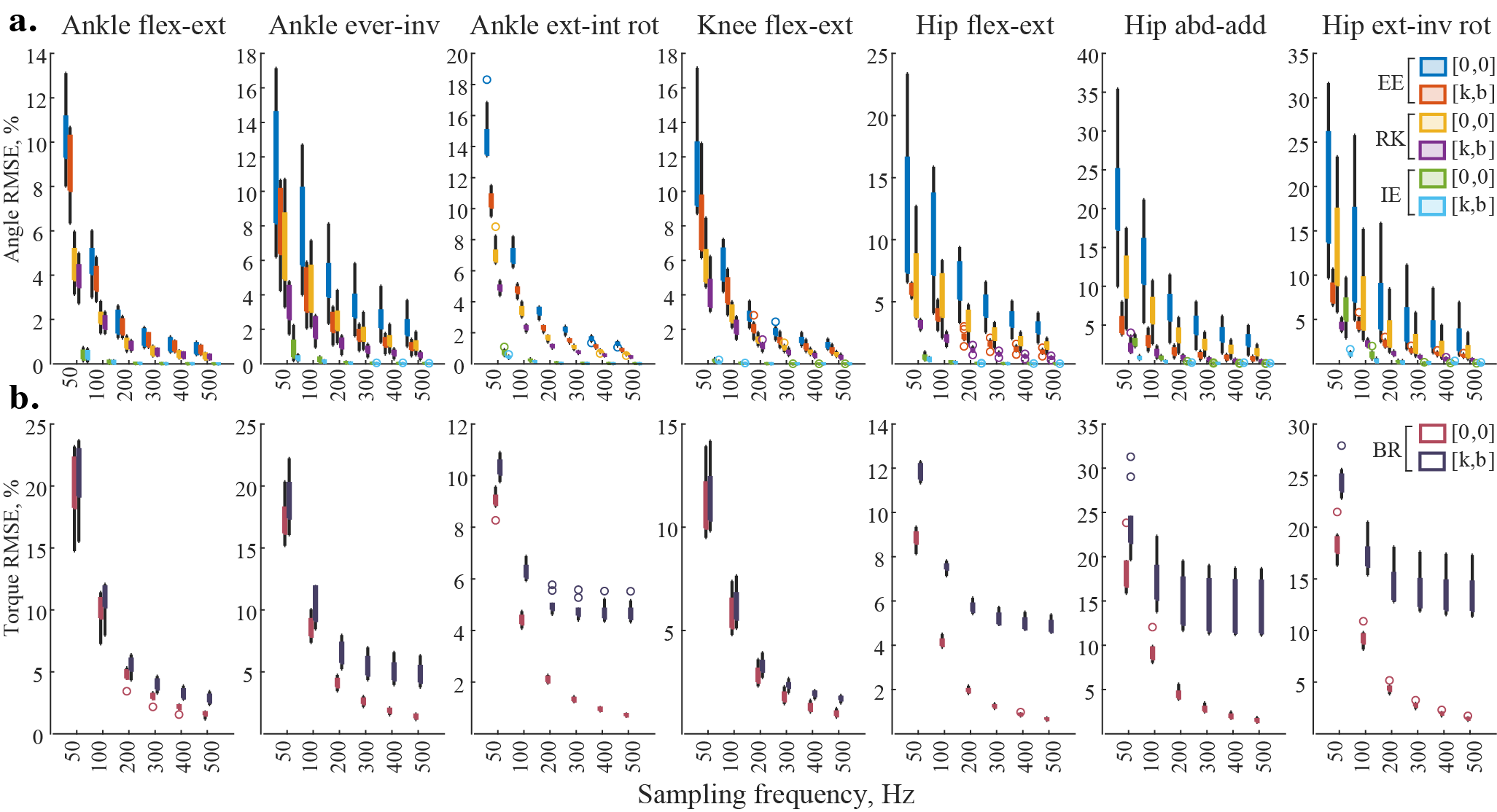


**S4 Fig.** Forward (A) and inverse (B) simulations accuracy are shown as a function of sampling rate, viscoelastic contribution, and numerical solver. The simulations in (A) were solved using numerical integrators: EE—explicit Euler method, RK—4th order Runge-Kutta method, and IE—implicit Euler method. Corresponding inverse simulations (B) were solved with a built-in recursive solver—BR. Labels [k,b] marked the error distributions obtained with optimal impedance.

Summary

S3 Table. Summary table. The forward dynamics problem was solved with explicit Euler—EE, implicit Euler— IE, and 4th order Runge-Kutta—RK methods at six sampling rates. The optimal solver and simulation rate pair is bolded. The inverse dynamics was solved at the same rates with a built-in recursive method—BR. The effects of impedance are listed for each configuration of simulation parameters.

| Problem | Solver | Sampling rate, Hz | Impedance |
| --- | --- | --- | --- |
| Forward dynamics | EE | 50  100  **200**  300  400  500 | necessary at low sampling rates |
|  | **IE** |  | unnecessary |
|  | RK |  | necessary at low sampling rates |
| Inverse dynamics | BR |  | effects are negligible at low sampling rates |
