# Supplementary material for: Does joint impedance improve dynamic leg simulations with explicit and implicit solvers?
Source: PLoS One. 2023 Jul 3;18(7):e0282130. doi: 10.1371/journal.pone.0282130 (PMC10317227; doi:10.1371/journal.pone.0282130)
Supplement: Attachment — Submitted filename: Response to Reviewers.docx [file pone.0282130.s006.docx]

Responses to editor and reviewers

Dear editor and reviewers,

We appreciate your efforts and contributions to our manuscript improving clarity and addressing most significant gaps. We detail our answers to the critiques below. The corresponding modified text was inserted in the manuscript and attached to the responses marked in *red* *italic*. All line numbers cited in our responses refer to the revised ‘clean’ version of our manuscript.

**Reviewer #1**

[Reviewer’s summary] The present study is well-written and provides useful information for researchers who are interested in dynamic simulation of human movement.

Thank you for this general assessment.

My main concern lies in Section D (lines 185-203). I am a bit confused about the "optimization" problem stated in this section. Did the authors actually solve any optimization problems to find the optimal k and b? Or they just found the minimal integrated kinematic and kinetic error (line 198) from 25 simulations across the ranges of k and b specified in lines 191-192? If the latter is the case, I won't recommend calling it an optimization problem since no actual searching is performed. It seems to me the authors have solved a sensitivity problem not an optimization problem.

The use of streamlined terminology is very important. The sensitivity and optimization terms are closely related, and we do recognize the need for clarification in our context. Our analysis varies impedance to assess kinematic and kinetic errors. While it is focused on the optimization of parameters for simulations with minimal errors, the range of search is narrowly defined. It is limited by the divergent effects on the two modalities of errors. We tend to agree with the reviewer that the use of iterative search for impedance is closer to the definition of a sensitivity problem and not an optimization problem. We have modified the text accordingly:

- *Lines 200-203: Passive joint impedance was described with the rotational stiffness and damping (k,b) used to stabilize joint DOFs in multibody simulations [42]. As expected, high impedance not only decreased angular errors but also increased torque errors. This, therefore, presented a sensitivity problem, which involved identifying optimal impedance values to reduce simulation errors.*

[Minor comments]

- Lines 84-85: Shouldn't it be "fail with the increase in the simulation time step"?

Thank you for noticing this error. It is corrected.

- *Lines 95-96: However, not surprisingly, all simulations fail with the* ***increase*** *in the simulation time step, which is a challenge for applications with low sampling rates.*
- Eqs (1) and (2): What does each superscript represent in these two equations? For example, what does [NxKxB] mean in Eq. (1)? Please clarify them in the revision.

Thank you for drawing our attention to this. We added a sentence to clarify that the superscripts denote dimensionality:

- *Lines 215-218: …* *The superscripts denote dimensionality. N is the number of swing phases; K is the number of stiffness coefficients; B is the number of damping coefficients; D is the number of DOFs; S is the number of solvers; F is the number of simulation rates; θ ^pp^ and τ ^pp^ are vectors containing joint range of motion and torque peak-to-peak values, respectively; and (k,b) is optimal stiffness and damping matrix.*
- Results section: To help readers better grasp the effects of various choices on the accuracy and stability of the simulation, I would suggest the authors providing a table in the revision to summarize the main findings.

Thank you for your suggestion. We have incorporated a summary table (S3 Table) into the Supplementary Information. This addition serves to emphasize the findings presented in Figure 2 (contrasting impacts of impedance on forward and inverse simulations) and the previously labeled Figure S2, now designated as Figure S4 (showing a favorable reduction in errors with addition of the impedance). The additional description of the table is added.

- Lines 219-220: Have the authors tried to simulate leg swing with factors that are not optimal? For instance, I will be curious to know the effect of the worst selection of the factors on the accuracy of kinematics and kinetics. Will the resulting kinematics and kinetics errors be much greater than 1% and 5%, respectively? Such information might be useful to researchers having difficulties accessing the optimal choices.

These proposed simulations were performed in our sensitivity analysis of impedance parameters. We examined kinematic and kinetic errors. The errors in kinematics and kinetics are represented in Figure 2. A new supplementary Figure S2 is added to compliment and detail these results for all other DOFs of the leg in swing. We have also added a new Figure S3 showing the impedances that result in above-threshold kinetic and kinematic errors with different numerical methods.

- Fig.2b: Is angle RMSE actually reported in degree? A RMSE of 5x10^-2 degrees seems very small to me. Just want to confirm it.

Yes, the reported RMSE is in degrees. The small values are attributed to the numerical accuracy of the implicit Euler method and, perhaps more importantly, to the fact that we tested relatively brief simulations—each swing phase had an average duration of about 450 ms. Our new supplementary figures (S2 and S3) should add clarity to the comparisons of the errors across the solvers. We also make a note of this in the legend of Figure S3:

- *Line 688-689: Notice a hundredfold difference in scale between values in Figs 2b and S2b.*
- Fig. 6: Please indicate the sign of each angle in the figure.

Thank you. We have updated the legend entries to reflect the directionality of each signal, with the first movement type corresponding to the negative direction on the vertical axis and the second movement type corresponding to the positive direction.

- *Lines 345-348: Abbreviations: f-e—flexion-extension; ab-ad—abduction-adduction; int-ext—internal-external rotation; ever-inv—eversion-inversion; in each legend entry, the first movement type corresponds to the negative direction on the vertical axis and the second movement type corresponds to the positive direction.*
- Fig. S2b: Only two legends are provided here but more than two colors are displayed. Please provide the legends for the other colors.

Thank you for the suggestion. To eliminate any possible confusion, we have added another legend entry to Figure S4b (former S2b). Additionally, we have modified the coloring scheme to avoid any similarities with the colors in Figure S4a. We included the information about the directionality in the legend:

- *Lines 692-697: S4 Fig. Forward (A) and inverse (B) simulations accuracy are shown as a function of sampling rate, viscoelastic contribution, and numerical solver. The simulations in (A) were solved using numerical integrators: EE—explicit Euler method, RK—4th order Runge-Kutta method, and IE—implicit Euler method. Corresponding inverse simulations (B) were solved with a built-in recursive solver—BR. Labels [k,b] marked the error distributions obtained with optimal impedance.*

**Reviewer #2**

[Reviewer’s summary] The paper proposed a biomechanical framework to test viscoelastic joint constraints and numerical solver types to decrease simulation errors at low sampling rates. Viscoelastic joint impedance to smooth the transient kinematic perturbations due to the large integration time step was used to increase simulation stability. The method was tested on a human bipedal model. The manuscript is well written and structured.

Thank you for your feedback and general enthusiasm about our work.

However, following suggestion may improve the quality of paper; Discussion section need to be improved.

We have done a deep revision of both formatting and text by improving clarity and composition throughout the manuscript. Moreover, we have added additional comprehensive details in a table and supplementary figures (see responses to Reviewer 1) that allow further comparisons to published studies (see added text below).

The authors have well described and explained the results but additionally proposed work should be compared with benchmark studies. Moreover English of the manuscript need to be improved as academic writing, …

Following your suggestion, we have expanded the Discussion in relation to the state-of-art formulations in the field, in addition to the previous focus on the MuJoCo engine. We have revised our discussion to include the comparisons of errors reported in other studies:

- *Lines 356-367: The simulation errors were small in forward (< 1%) and inverse (< 5%) simulations as computed with implicit Euler and built-in recursive methods, respectively (see also S4 Fig).* ***The inaccuracies in kinematics associated with explicit solvers matched values found in prior research on human locomotion [57], [58]****. Added viscoelastic impedance increased the precision of explicit simulations but, in general, decreased the physiological plausibility of the forces computed in inverse simulations.* ***Nevertheless, small errors could be tolerated as they do not surpass the inaccuracies observed in previous studies [7], [59] and result in increased integration accuracy with explicit methods, particularly when the frequency of simulations is low*** *(Fig 5). The implicit integration method outperformed all explicit methods and required no additional compensation from the impedance. Since explicit methods are standard in physics engines, the use of joint impedance could improve their numerical stability; however, implicit methods deliver the best results.*

**…** and formatting of the paper need to be reviewed as Figure numbering should be corrected, Fig 2A and 2B comes after Fig 3-4 and 5 in text citations.

Thank you for drawing our attention to this problem. We have removed the reference to Fig. 3-4 from the line 224 (former *line 209*) and the reference to Fig. 5 from the line 225 ( former *line 210*).
